# Binding of RNA by the Nucleoproteins of Influenza Viruses A and B

**DOI:** 10.3390/v8090247

**Published:** 2016-09-13

**Authors:** Alice Labaronne, Christopher Swale, Alexandre Monod, Guy Schoehn, Thibaut Crépin, Rob W. H. Ruigrok

**Affiliations:** 1 Institut de Biologie Structurale (IBS), University Grenoble Alpes, CEA, CNRS, 38044 Grenoble, France; alice.tissot@ibs.fr (A.L.); cswale@embl.fr (C.S.); alexandre.monod@orange.fr (A.M.); guy.schoehn@ibs.fr (G.S.); rob.ruigrok@ibs.fr (R.W.H.R.); 2 EMBL Grenoble Outstation, 71 Avenue des Martyrs, BP181, F-38042 Grenoble CEDEX 9, France

**Keywords:** influenza virus, nucleoprotein, RNA, oligomerization, assembly, ribonucleoprotein

## Abstract

This paper describes a biochemical study for making complexes between the nucleoprotein of influenza viruses A and B (A/NP and B/NP) and small RNAs (polyUC RNAs from 5 to 24 nucleotides (nt)), starting from monomeric proteins. We used negative stain electron microscopy, size exclusion chromatography-multi-angle laser light scattering (SEC-MALLS) analysis, and fluorescence anisotropy measurements to show how the NP-RNA complexes evolve. Both proteins make small oligomers with 24-nt RNAs, trimers for A/NP, and dimers, tetramers, and larger complexes for B/NP. With shorter RNAs, the affinities of NP are all in the same range at 50 mM NaCl, showing that the RNAs bind on the same site. The affinity of B/NP for a 24-nt RNA does not change with salt. However, the affinity of A/NP for a 24-nt RNA is lower at 150 and 300 mM NaCl, suggesting that the RNA binds to another site, either on the same protomer or on a neighbour protomer. For our fluorescence anisotropy experiments, we used 6-fluorescein amidite (FAM)-labelled RNAs. By using a (UC)_6_-FAM^3′^ RNA with 150 mM NaCl, we observed an interesting phenomenon that gives macromolecular complexes similar to the ribonucleoprotein particles purified from the viruses.

## 1. Introduction

Influenza belongs to the group of negative strand RNA viruses (NSVs). These viruses have a RNA genome that is coated with nucleoprotein (NP); the nucleoprotein–RNA complex (N–RNA) is the template for both transcription and replication by the viral polymerase. NP, viral RNA (vRNA), and polymerase constitute the ribonucleoparticle (RNP). Within the group of negative-strain RNA viruses, two genome organizations have been described: viruses with their entire genome made up of a single RNA molecule (non-segmented NSVs) and viruses with segmented genomes. The influenza viruses belong to the latter, with eight segments for types A and B.

To date, many X-ray structures of nucleoproteins bound to RNA have been published [1,2]. The first structures of the nucleoprotein–RNA complexes of non-segmented NSVs were these of vesicular stomatitis virus and rabies and showed that each nucleoprotein binds nine bases [3,4]. Thereafter, the structure of N–RNA of the respiratory syncytial virus showed seven bases bound to the nucleoprotein [5,6]. More recently, the structures from paramyxoviruses such as measles [7] and parainfluenza virus 5 [8] were solved. In both structures, six bases bind per nucleoprotein, confirming previous work based on biochemistry, electron microscopy (EM), and molecular virology [9,10,11]. The structures of the nucleoprotein–RNA complex from segmented RNA viruses are these of Lassa, binding six to seven bases [12,13], Rift Valley fever virus with seven bases [14], and Orthobunyaviruses with 11 bases [15,16,17,18,19]. Several structures of other segmented NSVs’ nucleoproteins have been obtained, including those of influenza, Ebola, and Borna viruses, but without RNA [20,21,22,23,24,25,26,27].

**Figure 1:**
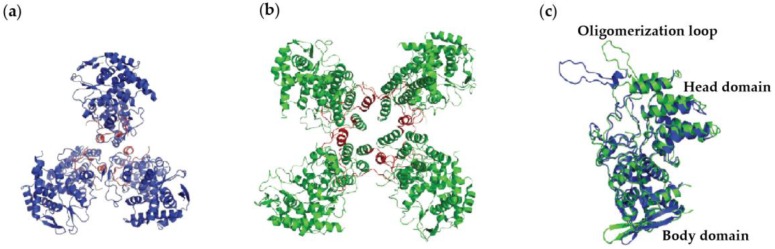
X-ray structures of influenza virus nucleoproteins. (**a**) Influenza A nucleoprotein (A/NP) (PDB ID: 2IQH) in blue forms a trimer, whereas (**b**) Influenza B nucleoprotein (B/NP) (PDB ID: 3TJ0) in green forms a tetramer. The oligomerization loops are coloured red on both (**a**) and (**b**). (**c**) Overlay of A/NP and B/NP. The overall folds of A/NP and B/NP are very similar with a head and a body domain. The root-mean-square deviation (r.m.s.d.) is 1.49 Å for 458 Cα. The exchange domains of the two structures point in different directions because they are bound to different neighbours.

For influenza virus, two structures of the nucleoprotein of influenza A (A/NP) and influenza B (B/NP) have been obtained: A/NP trimers [25,27] and its R416A monomeric mutant [28], and B/NP tetramers [24]. Figure 1 shows the structures of the A/NP trimer and B/NP tetramer. The way that this protein makes oligomers is through an oligomerization loop that goes from one protomer into its neighbour. The loop makes an angle from their head/body core, thus making either trimers or tetramers (Figure 1). Negative staining electron microscopy of NP from influenza A/PR/8/34 showed monomers and trimers and many larger oligomers, but no dimers [29]. Within this paper, we will show that B/NP can make dimers, probably because the angle of the loop allows dimers in contrast to A/NP. No structure of nucleoprotein–RNA complex exists for influenza virus. Early work using biochemistry and electron microscopy on RNPs showed that each protomer of nucleoprotein binds about 20 bases [30,31,32,33]. Later, Ortega et al. [34] reconstituted RNPs from NP, viral RNA polymerase, and several vRNAs with different sizes. It was found that the RNPs with 48 bases per pair of NPs amplify best. Hutchinson et al. [35] used mass spectrometry on purified A/WSN/33 virions and found that NP binds 26 bases (13,588 bases for 530 copies of NP per virion). However, the work on the nucleoprotein of infectious salmon anaemia virus (ISAV), a fish orthomyxovirus [36], suggested that NP only binds 12 bases, although the sizes of the RNPs are the same when compared to influenza A and B viruses. These authors suggested that only 12 bases bind in the potential RNA-binding groove, whereas the other 12 bases bind in another way to the nucleoprotein.

Several groups have measured the affinities between the influenza virus A/NP and B/NP towards RNA with different methods (Table 1). Most studies used wild-type or mutant recombinant protein, either His-tagged or tagged with a maltose-binding protein (MBP) tag that was removed before the measurements. Baudin et al. [37] used NP from influenza A/PR/8/34 virus. For both NPs from A and B viruses, most of the apparent dissociation constants (*K_d_*) were found to range from 4 to 70 nM, with experiments done at room temperature, 25 °C, and 37 °C. One test at 4 °C gave a 10-fold higher *K_d_* (380 nM). The monomeric mutants (R416A and E339A) bind RNA with much higher *K_d_*, between 1 and 10 µM [38]. The RNA-free recombinant or virus-purified NP displays a dynamic oligomeric behaviour, forming monomers, trimers/tetramers, and higher order oligomers with structures similar to real RNPs, depending on the protein concentration and salt content [29,39]. Tarus et al. [39] found that monomeric NP binds a 24-nucleotide (nt) RNA with a *K_d_* of around 40 nM, whereas the trimer of NP binds with an affinity of 24 nM. They also found that trimers remain trimeric when bound to RNA, whereas the monomer makes longer N–RNA complexes. This suggests that the trimers with RNA are blocked for making larger complexes.

In this paper, we show the effects of RNA binding on the oligomeric state of the NP from influenza viruses A and B, using size exclusion chromatography-multi-angle laser light scattering (SEC-MALLS), electron microscopy, and *K_d_* measurements. We wanted to define a condition to obtain a homogeneous NP monomer bound to RNA for X-ray crystallization, but we did not find such condition. This paper describes the problems in making such a complex between NP and RNA. However, we have observed an interesting phenomenon between influenza A nucleoprotein and a 12-nucleotide RNA labelled with a fluorescein probe, which form very large protein–RNA complexes, similar to RNPs purified from the virus.

## 2. Materials and Methods

### 2.1. Protein Expression and Purification of Influenza A and B NPs

The full-length NP genes of the H1N1 strain A/WSN/1933 and of the strain B/Memphis/13/03 were used. A/NP was cloned in pET22b (Novagen, Darmstadt, Germany) and B/NP in pETM11 (EMBL). *Escherichia coli* BL21 (DE3) cells were transformed with the resulting plasmids. Expression of the recombinant protein was induced by adding 0.3 mM isopropyl-β-d-thiogalactopyranoside (IPTG; Euromedex, Souffelweyersheim, France). Cultures were grown at 18 °C for 12 h and cells were collected by centrifugation at 4000 *g*, for 15 min at 4 °C. Pellets were resuspended and sonicated in lysis buffer (50 mM Tris-HCl pH 7.5, 300 mM NaCl, 1 M NDSB201 (Sigma, Saint-Quentin Fallavier, France), 5 mM β-mercaptoethanol (β-ME; Roth, Lagny-sur-Marne, France) containing a cOmplete™ protease inhibitor cocktail (Roche, Meylan, France). Purifications were performed at room temperature. Proteins were purified by Ni^2+^ affinity chromatography (Ni-NTA, Qiagen; Les Ulis, France) followed by a heparin column (GE-Healthcare, Dutscher, Brumath, France). Heparin elution fractions were dialyzed against 20 mM Tris-HCl pH 7.5 at 50 mM, 150 mM or 300 mM NaCl, and 5 mM β-ME. The last purification step was a size-exclusion chromatography using a Superdex increase S200 10/300 GL column (GE-Healthcare). The proteins were eluted in 50, 150, or 300 mM NaCl according to the experimental needs. Peak fractions were concentrated using a 10 kDa concentrator (Amicon, Dutscher, Brumath, France). The protein concentrations were determined using the extinction coefficients at 280 nm for A/NP ε = 55,537 M^−1^·cm^−1^ and for B/NP ε = 27,975 M^−1^·cm^−1^.

### 2.2. Fluorescence Anisotropy Measurements

Fluorescence anisotropy assays were performed on a Clariostar (BMG Labtech, Champigny-sur-Marne, France) microplate reader, fitted with polarization filters to measure fluorescence anisotropy. The binding assays were done in 384-well plates at room temperature (60 µL reaction volume) in 20 mM Tris-HCl pH 7.5, 50, 150, or 300 mM NaCl and 5 mM β-ME. Concentrations ranging from 0 to 3 µM of A/NP or B/NP were titrated into 5 nM ^5′^phosphate RNA labelled in 3′ with 6-fluorescein amidite (FAM). Five to 24 nt polyUC RNA (Integrated DNA Technologies, Coralville, IA, USA) were used. After subtracting the polarization values obtained for RNA alone, the mean value of three independent experiments were fitted to the standard binding equation:

(1) y = ((Bmax × xh)/(Kdh + xh))

assuming a single binding site with Hill slope (h) using Prism (Version 7, GraphPad, La Jolla, CA, USA). A control titration with A/NP and fluorescein alone was performed in order to confirm that the protein does not bind directly to fluorescein (Figure S4a). Bmax is the maximum specific binding in the same units as *y*.

### 2.3. Electron Microscopy

Samples (concentrations close to 0.05 mg·mL^−1^) were applied between a carbon and a mica layer. The carbon was then floated on the top of a heavy atom salt drop (2% (w/v) sodium silicotungstate, pH 7.0). The carbon film was covered by a copper grid. Both were fished using a small piece of paper and air dried before insertion in the electron microscope [43,44]. Charge-coupled Device (CCD) frames were taken with a T12 microscope (FEI, Hillsboro, OR, USA) operating at 120 kV and a nominal magnification of 45,000 times. The ^5′^phosphate-polyUC-FAM^3′^-NP complex was incubated overnight at room temperature using 100 µM of proteins in 20 mM Tris-HCl pH 7.5, 50, 150 or 300 mM NaCl and 5 mM β-ME and with a final ratio NP:RNA of 1:1. The dilutions for EM were performed with a buffer with the same salt concentration.

### 2.4. SEC-MALLS Experiments

SEC was performed with a Superdex increase S200 10/300 GL column (GE healthcare) equilibrated with 20 mM Tris-HCl, pH 7.5, 50, 150, or 300 mM NaCl, and 5 mM β-ME. Analytical runs were performed at 20 °C with a flow rate of 0.5 mL·min^−1^. Fifty microlitres of a sample between 3 and 5 mg·mL^−1^ were injected. ^5′^phosphate-polyUC-OH^3′^-NP complexes with a ratio NP:RNA of 1:1 were incubated for 1 h at room temperature before injection. MALLS detection was performed with a DAWN-HELEOS II detector (Wyatt Technology, Toulouse, France) using a laser emitting at 690 nm and protein concentration was measured on-line with the use of differential refractive-index measurements, with an Optilab T-rEX detector (Wyatt Technology) and a refractive-index increment (dn/dc) of 0.185 mL·g^−1^. Weight-average molar masses (Mw) were calculated with ASTRA (Wyatt Technology) as previously described [45].

## 3. Results

**Figure 2:**
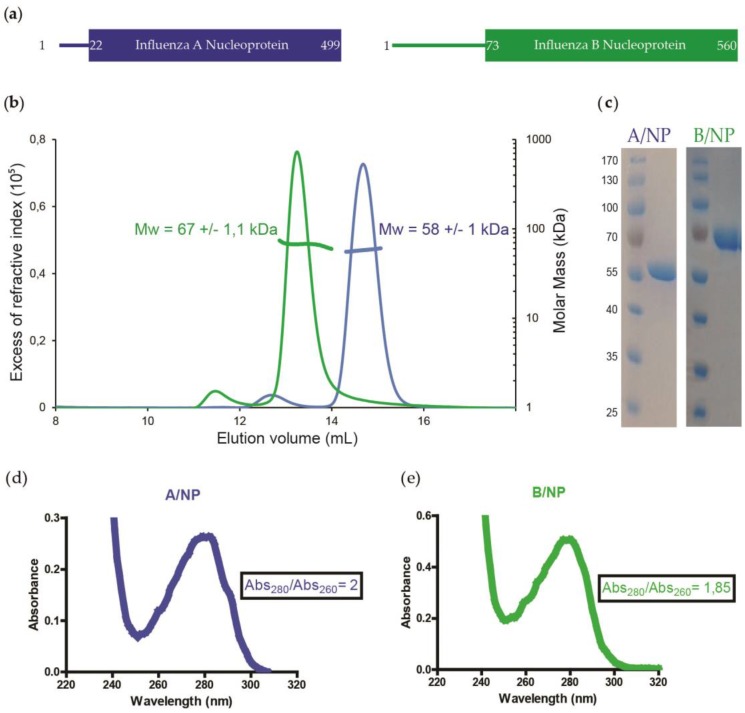
Homogeneous sample analysis of nucleoproteins of influenza A and B viruses. (**a**) Schematic representations of A/NP (blue) and B/NP (green). The A/NP presents a short 21 amino acid N-terminus, whereas B/NP has a longer N-tail of 72 amino acids; (**b**) Comparison of the size exclusion chromatography-multi-angle laser light scattering (SEC-MALLS) analysis of A/NP and B/NP at 50 mM NaCl. Experimental molecular weights, next to the peaks, are coherent with the expected masses of 58 and 65 kDa for A/NP and B/NP, respectively; (**c**) Coomassie blue-stained sodium dodecyl sulfate polyacrylamide gel electrophoresis (SDS-PAGE) gel of the purified A/NP and B/NP; Panels (**d**) and (**e**) are, respectively, the ultraviolet (UV) spectra of the A/NP and B/NP. Mw: molecular weight; Abs: absorbance.

The nucleoproteins of influenza viruses A and B were purified as monomers with only 5% of the protein forming larger oligomers (Figure 2b). The UV spectra of each sample showed no nucleic acid contamination. The B/NP is longer than A/NP because of a longer N-terminal tail (Figure 2a and Figure S1).

**Figure 3:**
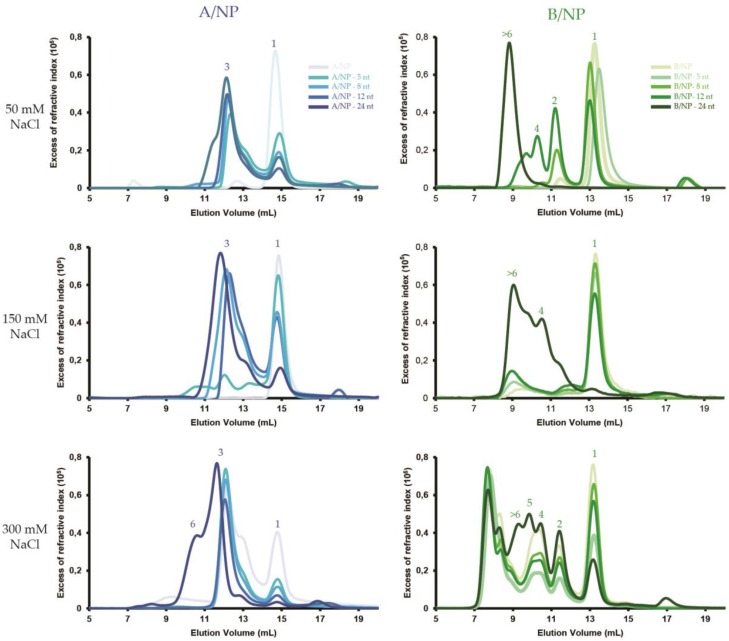
SEC-MALLS analysis of oligomeric states of the nucleoproteins of influenza A and B virus according to RNA length. SEC-MALLS analysis were performed on a Superdex 200 increase column equilibrated with 20 mM Tris-HCl pH 7.5 and different NaCl concentrations, from 50 to 300 mM. Protein is injected alone or in complex (ratio 1:1) with a short ^5′^phosphate-polyUC-OH^3′^ RNA from five to 24 nucleotides. Complexes are incubated 1 h at room temperature before injection. For each buffer condition, the relative proportion of oligomers eluted from the column gradually increased upon increasing RNA length. Peaks are labelled 1 for monomer, 2 for dimer, 3 for trimer, 4 for tetramer, 5 for pentamer, and >6 for larger than hexamer.

**Figure 4:**
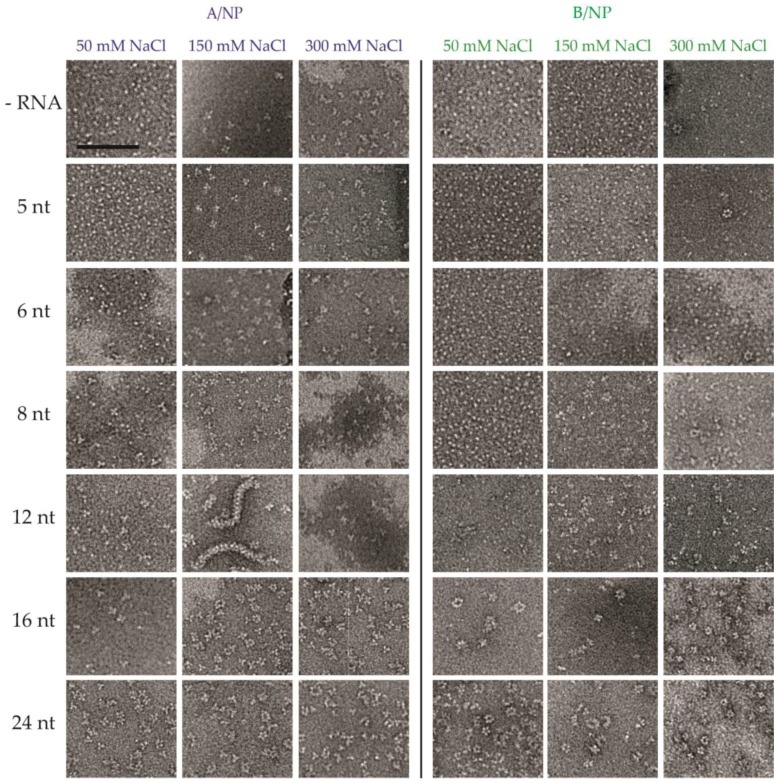
Electron microscopy of the nucleoproteins of influenza A and B viruses in the absence or presence of different RNA and different buffers. The A/NP or B/NP were incubated overnight at room temperature with or without ^5′^phosphate-polyUC-FAM^3′^ in 20 mM Tris-HCl pH 7.5, 5 mM β-mercaptoethanol (β-ME), and different NaCl concentrations from 50 to 300 mM. Samples were negatively stained with sodium silicotungstate. Electron micrographs show different oligomeric states depending on the salt concentration and the RNA used. The scale bar corresponds to 100 nm.

**Figure 5:**
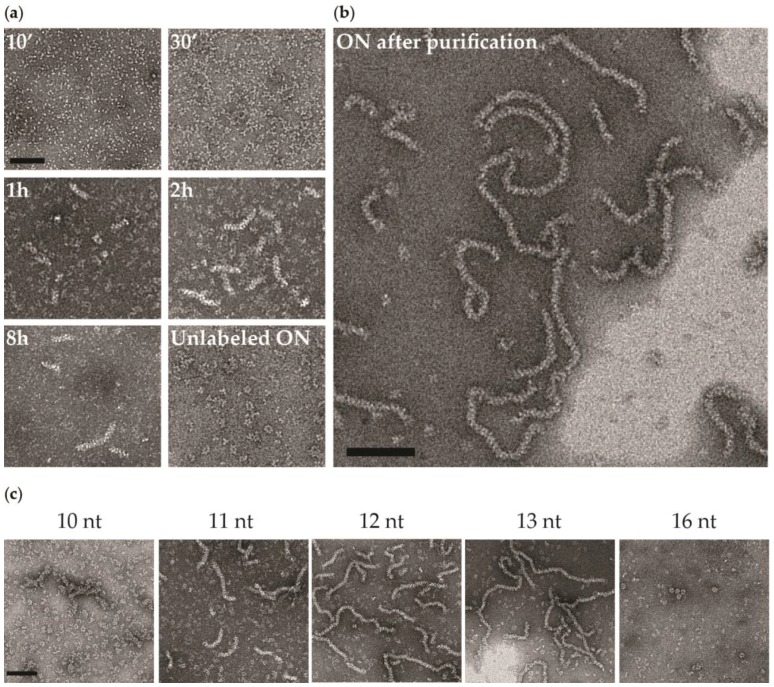
Ribonucleoprotein (RNP)-like species of influenza A virus nucleoprotein. Negative stain electron micrographs of A/NP with ^5′^phosphate-(UC)_6_-FAM^3′^. Complexes (ratio 1:1) were incubated at room temperature in 20 mM Tris-HCl pH 7.5, 150 mM NaCl, and 5 mM β-ME. Samples were negatively stained with sodium silicotungstate. (**a**) Kinetics of RNP-like formation with a 12-nucleotide RNA. Electron micrographs were taken at different times after incubation with several RNAs at room temperature. With an unlabelled RNA, no worm-like structure is observed, even overnight; (**b**) Worm-like structures after purification by centrifugation (10 min, 11,000× *g*, room temperature). The length of these species is random and they appear flexible. Similar to RNPs, these entities seem to be helical; (**c**) RNP-like species obtained with different RNA ^5′^phosphate-polyUC-FAM^3′^ lengths. With a 10-nt RNA, only a few short worm-like structures are observed. With 11 nt the worm-like structures are longer. With 12- and 13-nt RNAs, the worms are very similar with fewer trimers/tetramers in the background. The scale bars correspond to 100 nm. ON: overnight.

**Figure 6:**
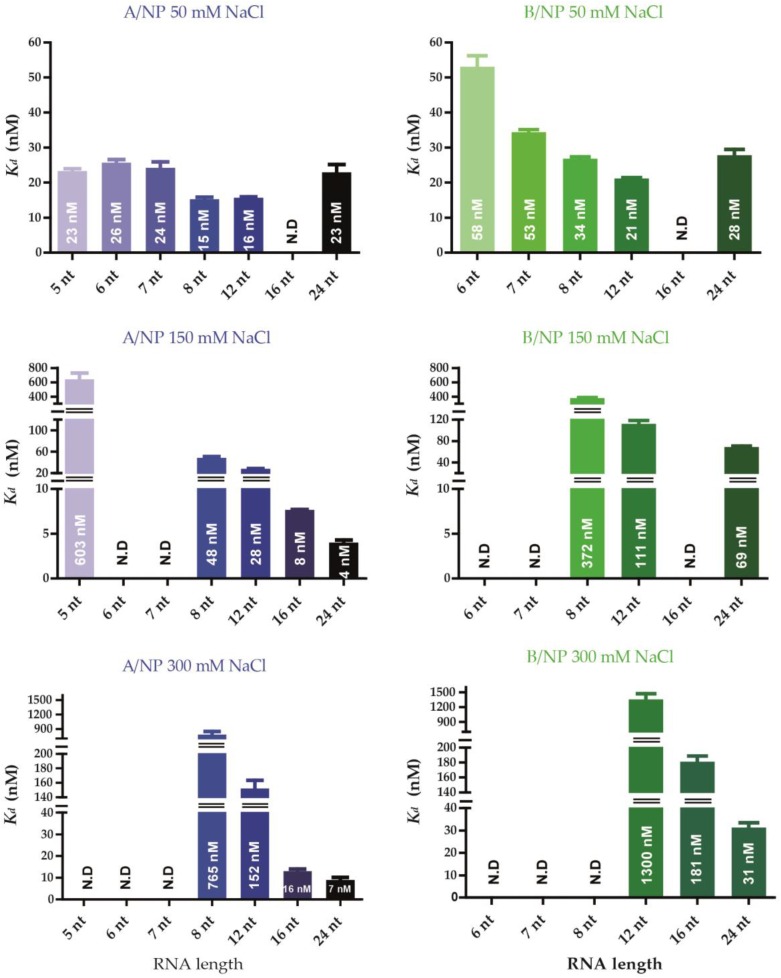
Affinities of both nucleoproteins of influenza A and B viruses for different RNAs. All the fluorescence anisotropy experiments were performed in triplicate in 20 mM Tris-HCl pH 7.5, 5 mM β-ME, and different NaCl concentrations (from 50 to 300 mM). The titration of NP was done against ^5′^phosphate-polyUC-FAM^3′^ RNAs ranging from 5 to 24 nt. The mixes were incubated 5 min at room temperature. Curves are shown in Figure S4. N.D: non-determined.

In order to assess the influence of both salt and RNA size on the binding of monomeric NP, a series of salt conditions (50, 150 and 300 mM NaCl) were tested against size-incremented polyUC RNA from 5 to 24 nt. The experiments were performed using SEC-MALLS (Figure 3 and Figure S2a,b), negative staining EM (Figure 4 and Figure 5) and fluorescence anisotropy (Figure 6 and Figure S4). A ^5′^phosphate-polyUC-OH^3′^ was used for the MALLS experiments and ^5′^phosphate-polyUC-FAM^3′^ for the negative staining EM and for the fluorescence anisotropy experiments.

### 3.1. SEC-MALLS

All the SEC-MALLS curves of A/NP and B/NP at 50, 150, and 300 mM NaCl are shown in Figure 3 (Figure S2a,b correspond to individual curves with the respective masses). For A/NP without RNA, the protein is mainly monomeric at 50 and 150 mM NaCl. When NP binds to RNA, the NP–RNA complexes form trimers rapidly. At 300 mM NaCl, there are only a few monomers and most of the protein makes trimers, with or without RNA. Only with a 24-nt RNA do the resulting complexes make larger structure entities. The behaviour of B/NP is quite different since it remains monomeric at 50 and 150 mM NaCl without RNA. With the RNA at 50 mM NaCl, dimers are obtained with 5- and 8-nt RNAs and then tetramers and much bigger entities with longer RNAs. At 150 mM NaCl, all complexes remain monomeric, except when a 24-nt RNA is added, which results in the formation of tetramers and larger complexes. At 300 mM the peak corresponding to the monomer becomes smaller with longer RNAs and we observe clear peaks corresponding to dimers, tetramers, pentamers, and several larger complexes, larger than those of A/NP with RNA, but not trimers.

### 3.2. Negative Staining EM

For these experiments different RNA molecules were used with a different time of incubation, overnight for EM instead of 1 h as for SEC-MALLS (Figure 4). For A/NP, at 50 mM NaCl, without RNA and with a 5-nt RNA, we observe monomers and with longer RNAs we see mainly trimers, even with 24-nt RNA. These data are coherent with SEC-MALLS results. At 150 and 300 mM NaCl, mainly trimers are observed in all cases, except when including the 12-nt RNA at 150 mM NaCl (this will be discussed later). For B/NP, at 50 mM only monomers are observed until the 12-nt RNA and with longer RNAs we see oligomers larger than with A/NP. At 150 and 300 mM, we observe monomers when RNAs of up to 6-nt are added, and with longer RNAs we see a mixture of dimers, tetramers, pentamers, and larger complexes making rings and “curly strings” [29].

In the case of A/NP, but not with B/NP, using a 12-nt ^5′^phosphate-(UC)_6_-FAM^3′^, we can observe oligomeric entities extremely different from all the other conditions. Figure 5 shows a time course of the sample by negative staining EM. Ten minutes after the preparation, the protein looks still monomeric and becomes trimeric after 30 min, then forming short rods. With time, the rods become longer and after one night at room temperature most of the protein has formed long worm-like entities similar to RNPs with a few remaining trimers. However, with a 12-nt ^5′^phosphate-(UC)_6_ the sample shows mainly trimers and a few larger complexes but no rods or worm-like structures. This means that the 6-fluorescein amidite at the 3′-end of the RNA provokes the reorganization of the NP–RNA complexes to these RNP-like entities. We found that the worm-like structures appear only at 150 mM NaCl, but not at 50 mM or 300 mM NaCl. This phenomenon is observed with fluorescein-labelled RNA ranging from 10 to 13 nt but not with 8 or 16 nt (Figure 4). Even if using 10, 11, and 13 nt RNAs gives similar results, the best homogeneous preparation is obtained with 12 nt.

### 3.3. Fluorescence Anisotropy Measurements

All the fits were good with R^2^ values above 0.98 (Figure S4c). Affinities between NP and RNA were measured using fluorescence anisotropy after 10 min incubation. From then on, the signal did not change. Figure 6 summarises the values of the *K_d_* in histograms and all the affinities are detailed in Figure S4b,c. In all conditions, A/NP binds RNA with a higher affinity than B/NP. For A/NP at 50 mM NaCl, all the strongest affinities are obtained with 8- and 12-nt RNAs. For 150 and 300 mM NaCl the affinities for the smaller RNAs, between 5 and 12 nt RNAs are much lower than with 50 mM NaCl and at 300 mM NaCl; the 5-nt RNA does not bind the protein. However, the affinity with a 24-nt RNA is enhanced at higher salt. For B/NP we see the same trends as with A/NP, although the RNA with 24 nt RNA does not bind better at a higher salt concentration.

## 4. Discussion

Using purified recombinant proteins without phosphorylation and with short RNAs, the experiments provide a rationale towards reconstructing viral-like nucleoprotein–RNA complexes. Nucleoproteins from influenza A and B were characterized and we found few biochemical differences between the two proteins.

Both proteins are monomeric at 50 and 150 mM NaCl. Both undergo a time-dependent oligomerization process with RNA, higher salt, and increasing protein concentration [25,27,29,39]. At 300 mM NaCl, both proteins are monomeric and form small oligomers. The experiment shown in Figure 5 and the results obtained by fluorescence anisotropy suggest that the RNA is rapidly bound on a monomeric protein and then, through a slower process, forms oligomers, at least in solution with 50 and 150 mM NaCl.

### 4.1. Nucleoprotein of A

**Figure 7:**
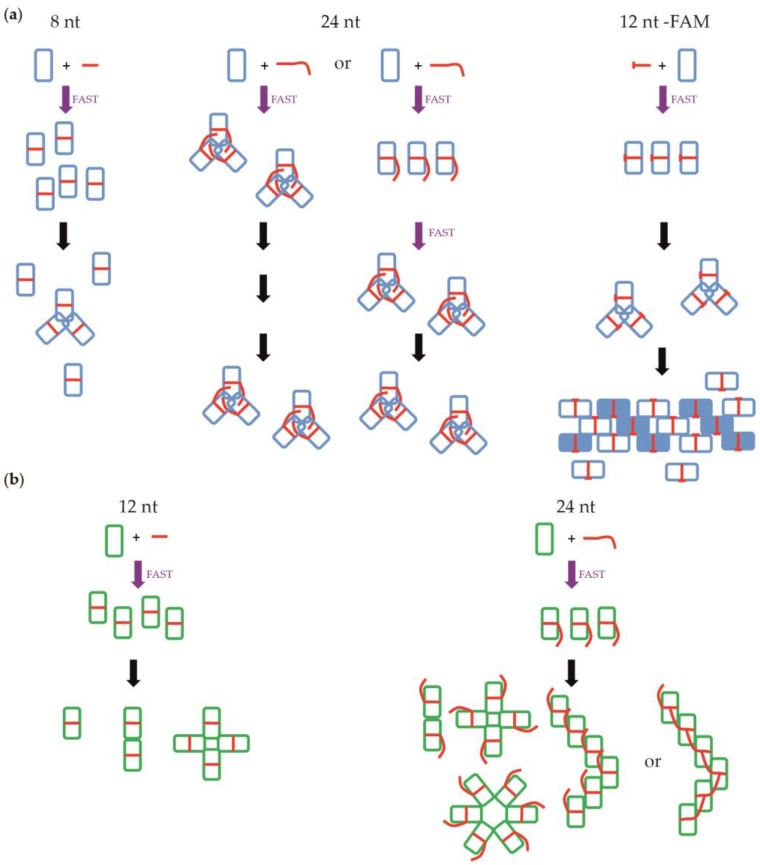
Schematic representation of the oligomerization of influenza A and B viruses’ nucleoproteins depending on the size of the RNA molecules at 150 mM NaCl. Panels (**a**) and (**b**) are for A/NP and B/NP, respectively.

At 50 mM NaCl we found that NP remains monomeric with small RNAs (up to 6 nt; Figure 7a). With 8 nt and longer RNA molecules, the monomers bind the RNA and then make trimers. At 150 mM NaCl, the monomers bind the RNA and then make trimers and larger complexes with 24-nt RNA. At 300 mM NaCl, part of the protein is already trimeric, so the binding of the RNA is to monomers and trimers and then all the complexes become trimers, making larger complexes with 24-nt RNA. The affinities at 50 mM NaCl are all similar, suggesting that the RNA is bound at the same site on the protein although 12-nt RNA binds slightly better than a 24-nt RNA (Figure 6). With more salt, shorter RNAs bind much less well but 24-nt RNA binds better than at 50 mM NaCl. At 300 mM NaCl, the trend is even stronger. Because the binding with a 24-nt RNA is stronger, this suggests that the RNA binding site is different for a 24-nt RNA at 150 and 300 mM NaCl, probably because under these conditions the RNA binds trimers and not monomers. Because the affinity of the RNA for the trimer is so strong, the trimers may have a problem disassembling and forming longer complexes.

### 4.2. Nucleoprotein of B

For NP of influenza B, we observed similar results (Figure 7b). The only differences are that NP stays monomeric with 12-nt RNA at 50 mM NaCl. With 6-nt RNA at 150 mM and 300 mM NaCl, we still observed monomers. However, we did not obtain trimers but dimers and tetramers, as seen also in Figure 3 with SEC-MALLS experiments. Using a 24-nt RNA, the complexes are larger than those from A/NP. The affinities measurements have shown that the 24-nt RNA binds with a lower affinity than the 12-nt RNA. However, the affinity towards the 24-nt RNA remains comparable at all tested salt concentrations. This suggests that the initial binding of the 24-nt RNA is always on the monomer and not to another binding site.

Three structures of A/NP [25,27,28] and one of B/NP [24] were determined previously. In A/NP crystal structures, the protein is always organized as trimers, whereas it forms tetramers within crystals of B/NP. This difference can be explained by the orientation of the oligomerization loop, which is not the same, leading to different oligomeric forms. The trimeric A/NP seems to be in a more closed conformation than the B/NP tetramer, which appears to be in a more open conformation. Maybe the differences in the affinities observed for A/NP and B/NP for RNA result from the oligomer states. Moreover, the sequence alignments show that the C-terminus of B/NP contains two aspartic acids that are not present in A/NP. This C-terminus might bind in the basic RNA groove and then keep B/NP in a monomeric form with longer RNA than A/NP.

### 4.3. NP of Influenza A with ^5′^Phosphate-(UC)_6_-FAM^3′^

Many studies use “FAM” for fluorescence anisotropy to measure affinities between proteins and RNAs. Zheng and co-workers [36] have used the same method for the binding of NP of infectious salmon anaemia virus with a ^5′^fluorescein labelled-(UC)_6_. Figure 5 shows that the RNA binds to the monomer and then the NP-RNA makes trimers, larger complexes, and flexible worm-like structures. These structures have the same diameter as the RNPs purified from influenza virus particles [29] and show fine lines, suggesting that these structures are helical. They are only obtained with fluorescein-labelled RNA. Without the fluorescein, NP assembles as trimers and does not generate these longer worm-like structures (Figure 5). With shorter RNAs, these structures are not observed either, probably because the NP-RNA remains monomeric or the reaction is too slow to be observed. With longer RNAs (≥16 nt RNA), the same is observed, maybe because the NP-RNA makes trimers rapidly and stops the growth of these worm-like structures. There is also an effect of the salt concentration: at 50 mM NaCl, the complexes remain monomeric, whereas at 300 mM they make trimers. Figure 6 suggests that the 12-nt RNA binds to the monomer at 50 mM but the 24-nt RNA could be too long for a monomer. The structure of the monomeric A/NP-R416A [28] is different from the trimer [27]. One side of the monomer has the C-terminus bound to the RNA binding site, changing the charge of the RNA groove. Zheng et al. concluded that each monomer of NP of ISAV binds about 12 bases but the RNA in the RNPs binds additional parts of the proteins [36]. In our experiments, only the monomeric NP with a ^5′^phosphate-(UC)_6_-FAM^3′^ can form worm-like structures. However, it is still to be determined why the labelled RNA makes these interesting entities that are not observed with the unlabelled RNA. However, interestingly, we also show by MALLS that A/NP and B/NP, in complex with labelled RNA, have a tendency to form higher oligomeric species at 150 mM NaCl compared to RNA without FAM (Figure S3). Fluorescein may thus have a role in the oligomerization process even if it does not bind NP directly (Figure S4a).

## 5. Conclusions

In this paper, we tried to understand the oligomerization process of A/NP and B/NP with respect to the salt concentration and the RNA length. Our results show many differences between the two proteins but it remains difficult to explain these without crystal structures of NP with RNA. However, we show that to crystallise the protein in the presence of RNA, it is necessary to find a compromise between the salt concentration and the RNA length. An unlabelled 12-nt RNA could be a good compromise between these two criteria because it binds strongly to the protein and is not too long to interfere with the crystallisation process. We also show that it is possible to reconstitute an RNP-like structure using recombinant NP in the absence of the RNA polymerase. We cannot yet explain why we can only have this species with a labelled RNA but it is a very interesting tool to start further investigations towards better understanding the interaction between NP and RNA.

## Figures and Tables

**Table 1:** Affinities of the nucleoproteins of influenza A and B for different RNAs. This table summarizes the results of affinity measurements from different studies. These studies used protein from different strains (A/WSN/1933; A/PR/8/34; A/HK/483/97(H5N1); B/Panama/45/90). Proteins are recombinant protein or directly purified from the virus. Recombinant proteins are MBP- or His-tagged. The MBP tag is cleaved after the first purification step. Different techniques were used to measure the affinities between nucleoprotein (NP) and RNA: filter binding assay (FBA), surface plasmon resonance (SPR), and polarization of fluorescence (Fluo-pol). Experiments were done at different temperatures, with different NaCl concentrations (from 100 to 300 mM) and with different RNAs (from eight to 890 nucleotides).

Virus	Protein	Method/Temp/[NaCl]	RNA	RNA Size (nt)	*K_d_* (nM)	Reference
***Influenza A***						
PR/8	virus	FBA/37 °C/100 mM	segment 8	890 nt	23	[37]
PR/8	virus	FBA/37 °C/100 mM	dsRNA	ds 20 nt	71	[37]
PR/8	(MBP)-NP ^1^	FBA/100 mM (KCl)	panhandle	178 nt	16	[40]
PR/8	His-NP	FBA/4 °C/100 mM	panhandle	81 nt	380	[41]
WSN	His-NP ^2^	SPR/25 °C/300 mM	^5′^biotin-RNA	24 nt	30	[39]
WSN	NP-His ^2^	SPR/25 °C/300 mM	^5′^biotin-RNA	24 nt	41	[39]
WSN	His-NP	SPR/25 °C/300 mM	^5′^biotin-RNA	24 nt	14	[39]
WSN	NP-His	SPR/25 °C/300 mM	^5′^biotin-poly	8 nt	70	[39]
WSN	His-NP	SPR/25 °C/300 mM	^5′^biotin-RNA	40 nt	17	[39]
H5N1	(MBP)-NP ^1^	SPR/25 °C/100 mM	^2′^O-methylated	24 nt	23	[25]
H5N1	(MBP)-NP ^1^	SPR/25 °C	^2′^O-methylated	24 nt	16	[38]
WSN	(MBP)-NP ^1^	Fluo-pol/RT/200 mM	^5′^fluo-RNA	20 nt	3.6	[42]
***Influenza A mutants***						
PR/8	E339A	FBA/4 °C/100 mM	panhandle	81 nt	1600	[41]
PR/8	R416A	FBA/4 °C/100 mM	panhandle	81 nt	2600	[41]
WSN	R416A	SPR/25 °C/300 mM	^5′^biotin-RNA	24 nt	10,000	[39]
H5N1	E339A	SPR/25 °C	^2′^O-methylated	24 nt	858	[38]
H5N1	R416A	SPR/25 °C	^2′^O-methylated	24 nt	975	[38]
WSN	Δ402-429	Fluo-pol/RT/200 mM	^5′^fluo-RNA	20 nt	1.7	[42]
***Influenza B***						
B	MBP-NP	SPR/25 °C/150 mM	^2′^O-methylated	24 nt	13	[24]

^1^ The parentheses indicate that the MBP-Tag has been removed for the *K_d_* measurements. ^2^ Monomeric nucleoprotein. Temp: temperature; PR/8: influenza A/PR/8/34; WSN: influenza A/WSN/1933; H5N1: A/HK/483/97(H5N1); B: influenza B/Panama/45/90; MBP: maltose-binding protein; NP: nucleoprotein.

## Supplementary Materials

The following are available online at www.mdpi.com/1999-4915/8/9/247/s1, Figure S1: Sequence alignment of the nucleoproteins of influenza A and B viruses; Figure S2a: Oligomeric states of influenza A virus nucleoprotein according to RNA length by size exclusion chromatography-multi-angle laser light scattering (SEC-MALLS) analysis; Figure S2b: Oligomeric states of influenza B virus nucleoprotein according to RNA length by SEC-MALLS analysis; Figure S3: Oligomeric state of nucleoproteins of influenza A and B viruses using polyUC-FAM^3′^ RNAs by SEC-MALLS analysis; Figure S4: Titration measurements against RNAs by fluorescence anisotropy. References [46,47] are cited in the supplementary materials.
