# Supplementary material for: Binding of RNA by the Nucleoproteins of Influenza Viruses A and B
Source: Viruses. 2016 Sep 13;8(9):247. doi: 10.3390/v8090247 (PMC5035961; doi:10.3390/v8090247)
Supplement: Supplementary file 1 [file viruses-08-00247-s001.pdf]

Supplementary Materials: Binding of RNA by the Nucleoproteins of Influenza Viruses A and B

Alice Labaronne, Christopher Swale, Alexandre Monod, Guy Schoehn, Thibaut Crépin and Rob W. H. Ruigrok

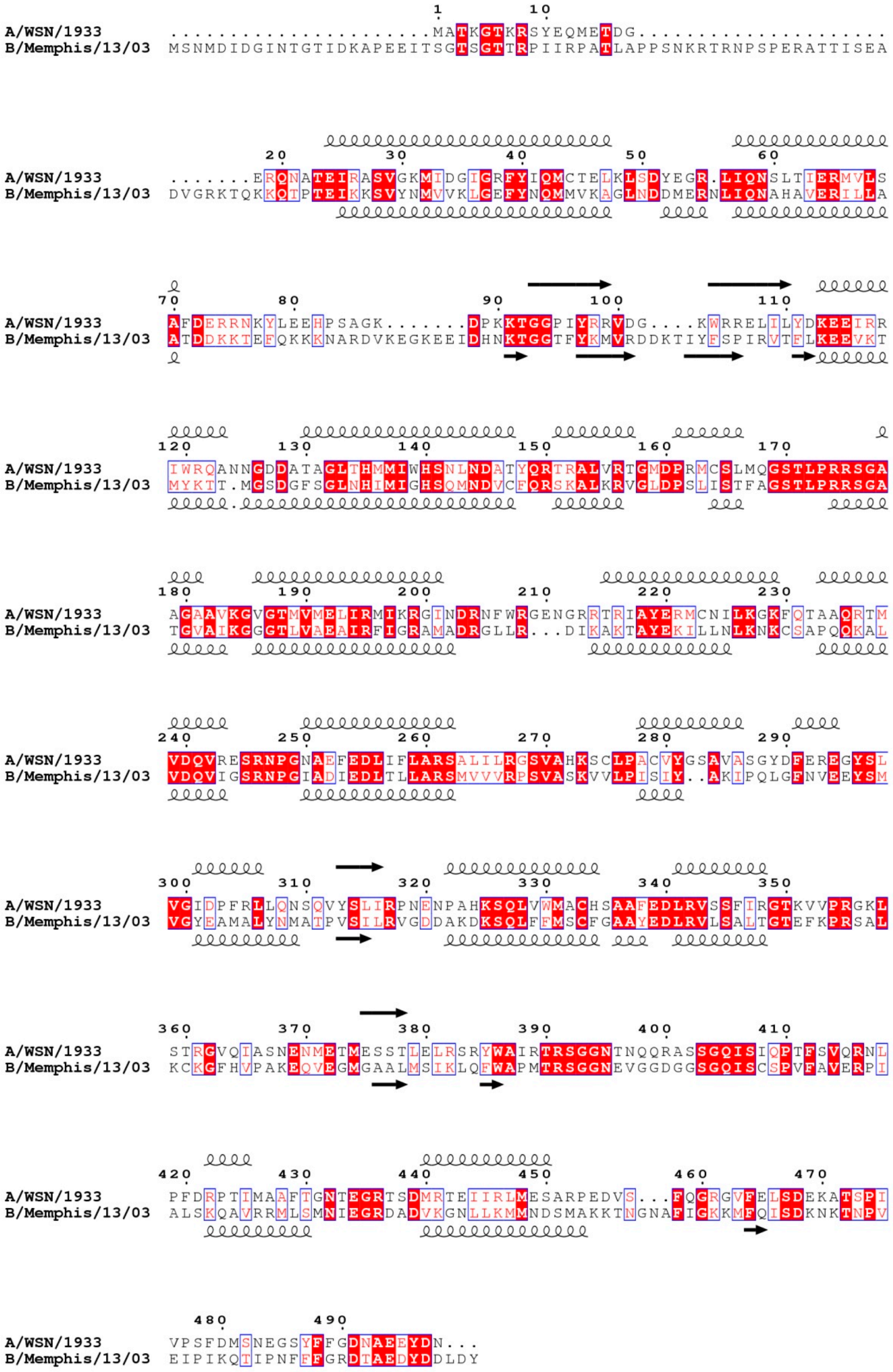

Figure S1. Sequence alignment of the nucleoproteins of influenza A and B viruses. The alignment has been made with the sequences of the two human influenza A and influenza B viruses used for this work: A/WSN/1933 (UniProtKB access number: Q1I2B5) and B/Memphis/13/03 (UniProtKB access number: Q5V8X3). The secondary sequences elements shown over and below the sequences alignment correspond to A/NP, A/WSN/1933 (PDB ID: 2IQH) and B/NP, B/Managua/4577.01/2008 (PDB ID: 3TJ0). The sequence identity between these two B/NP is 98.6%. The alignment has been done using Clustal W [46] and drawn with ESPript [47].

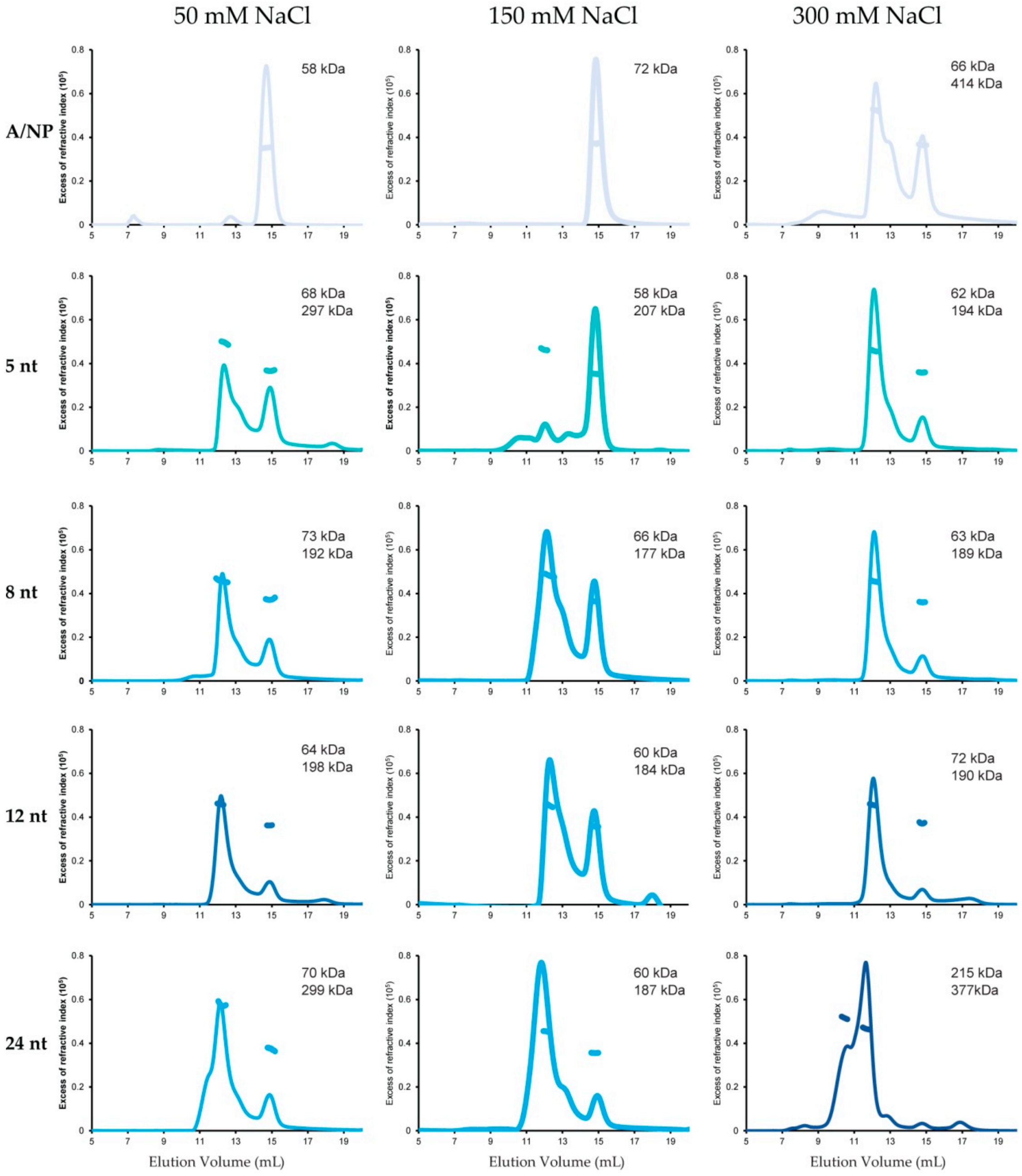

Figure S2a. Oligomeric states of influenza A virus nucleoprotein according to RNA length by size exclusion chromatography-multi-angle laser light scattering (SEC-MALLS) analysis. SEC-MALLS analysis were performed on a Superdex 200 increase 10/300 GL column equilibrated with 20 mM Tris-HCl pH 7.5 and different concentrations of NaCl (from 50 to 300 mM). Protein was injected alone or in complex (ratio 1:1) with short $^{5'}$phosphate-polyUC-OH$^{3'}$ RNAs (from 5 to 24 nt). Complexes were incubated 1 h at room temperature before injection. SEC-MALLS chromatograms are represented with the refractive index as a back trace and molecular weights are estimated at the left.

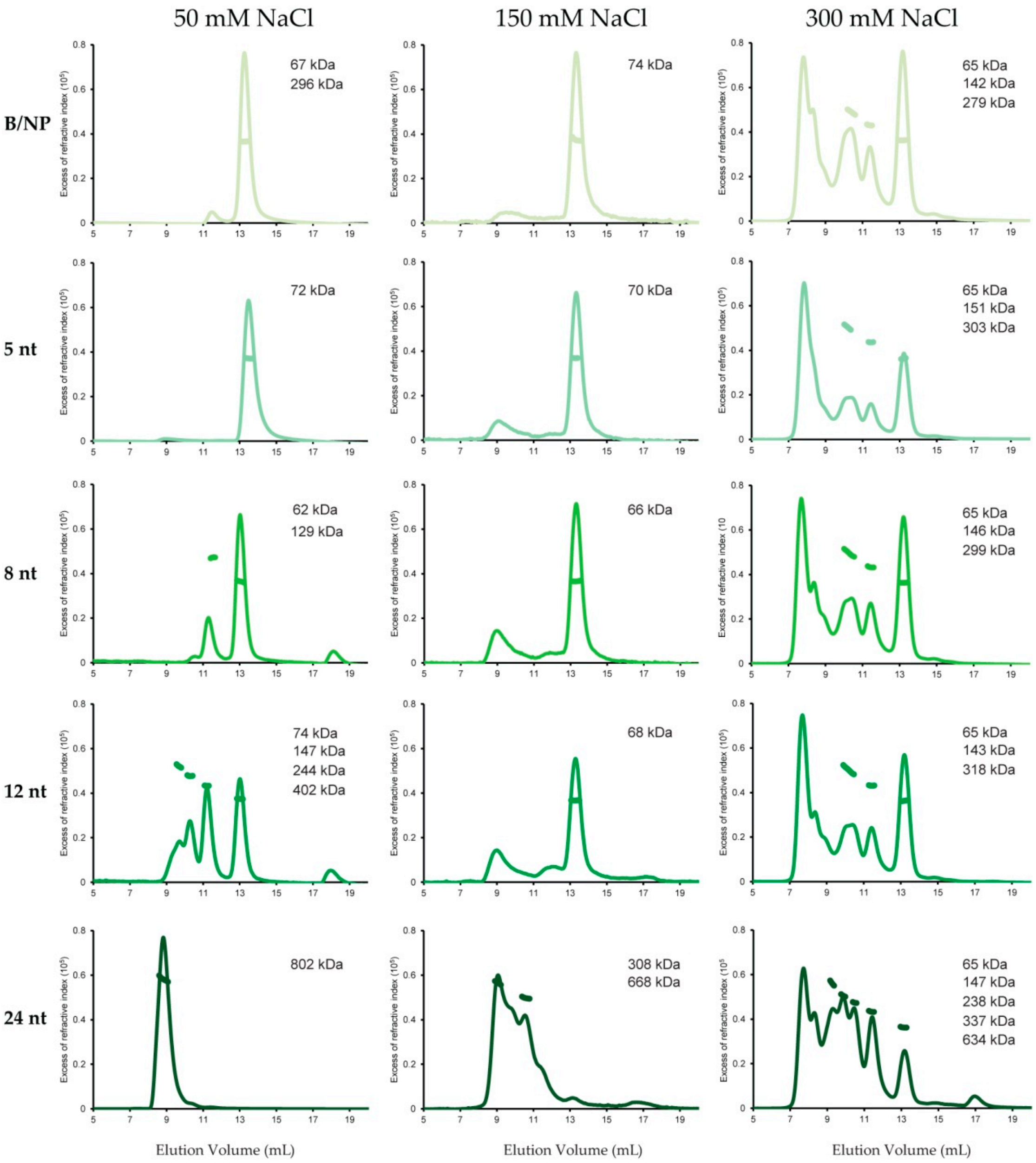

Figure S2b. Oligomeric states of influenza B virus nucleoprotein according to RNA length by SEC-MALLS analysis. SEC-MALLS analysis were performed on a Superdex 200 increase 10/300 GL column equilibrated with 20 mM Tris-HCl pH 7.5 and different concentrations of NaCl (from 50 to 300 mM). Protein was injected alone or in complex (ratio 1:1) with short $^{5'}$phosphate-polyUC-OH$^{3'}$ RNAs (from 5 to 24 nt). Complexes were incubated 1 h at room temperature before injection. SEC-MALLS chromatograms are represented with the refractive index as a back trace and molecular weights are estimated at the left.

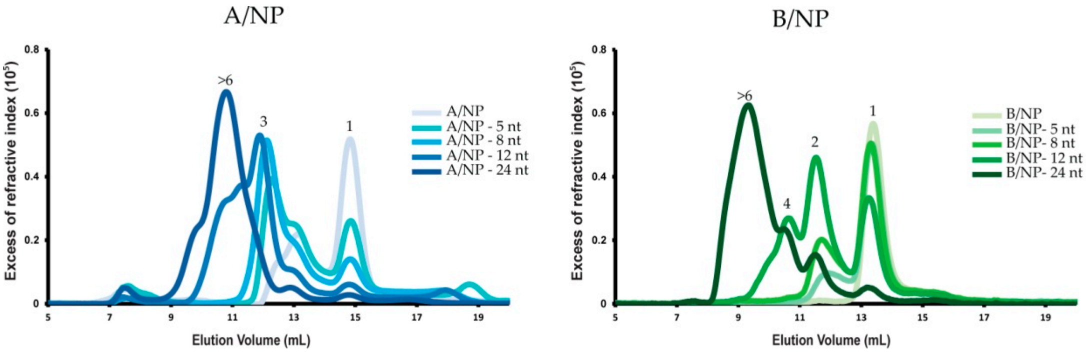

Figure S3. Oligomeric state of nucleoproteins of influenza A and B viruses using polyUC-FAM$^{3'}$ RNAs by SEC-MALLS analysis. SEC-MALLS analysis were performed on a Superdex 200 increase 10/300 GL column equilibrated with 20 mM Tris-HCl pH 7.5 at 150 mM NaCl. The protein was injected alone or in complex (ratio 1:1) with a single short $^{5'}$phosphate-polyUC-FAM$^{3'}$ RNA (5 to 24 nucleotides). The complexes were incubated 1 h at room temperature before injection. Peaks are labeled 1 for monomer, 2 for dimer, 3 for trimer, 4 for tetramer and 6 for hexamer.

(**a**)

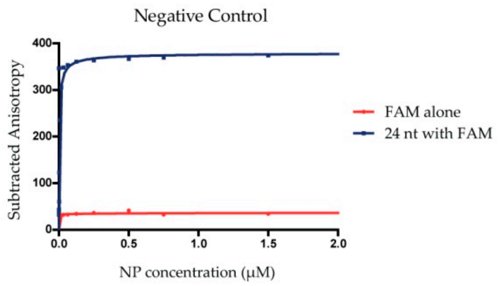

(**b**)

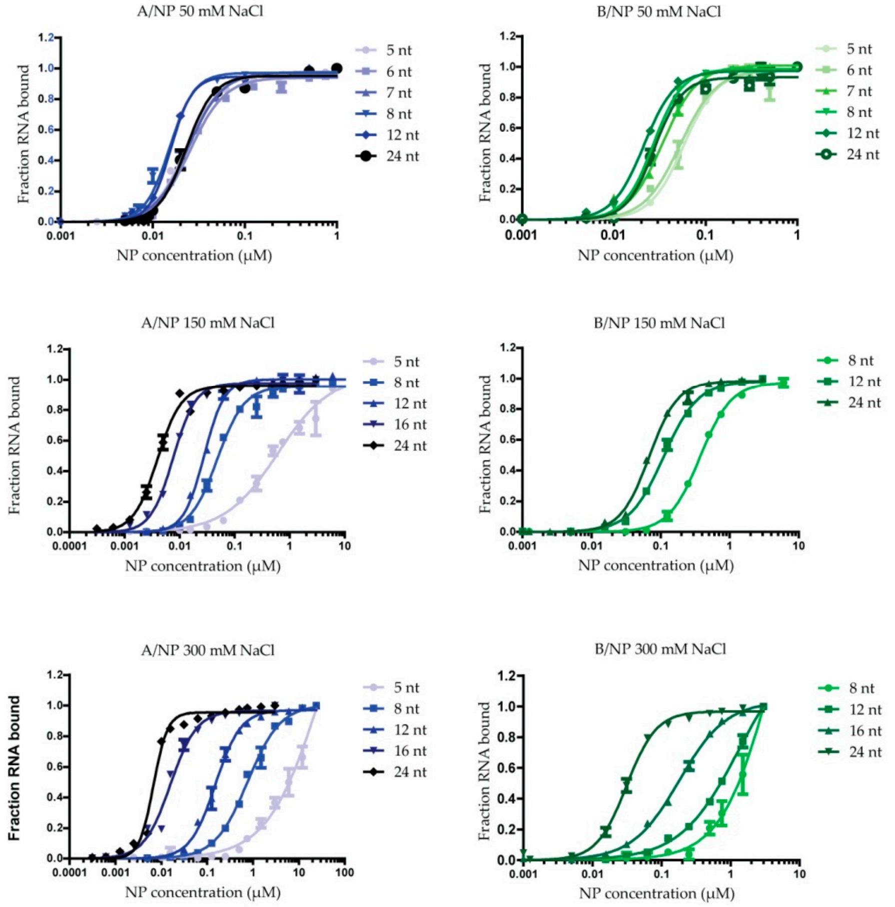

(**c**)

	50 mM NaCl		150 mM NaCl		300 mM NaCl	
**RNA**	K_d (nM)	R^2	K_d (nM)	R^2	K_d (nM)	R^2
**5 nt**	23 ± 0.8	0.9952	603 ± 93	0.9736		
**6 nt**	26 ± 0.4	0.9923				
**7 nt**	24 ± 0.5	0.9943				
**8 nt**	15 ± 0.8	0.9852	48 ± 2	0.9890	765 ± 40	0.9925
**12 nt**	16 ± 0.8	0.9908	28 ± 0.8	0.9925	151 ± 8	0.9899
**16 nt**			8 ± 0.2	0.9969	16 ± 0.8	0.9874
**24 nt**	23 ± 0.6	0.9917	4 ± 0.2	0.9873	7 ± 0.2	0.9917

	50 mM NaCl		150 mM NaCl		300 mM NaCl	
**RNA**	K_d (nM)	R^2	K_d (nM)	R^2	K_d (nM)	R^2
**5 nt**	58 ± 0.8	0.9978				
**6 nt**	53 ± 3	0.9748				
**7 nt**	34 ± 0.8	0.9919				
**8 nt**	27 ± 0.4	0.9985	372 ± 8	0.9973		
**12 nt**	21 ± 0.5	0.9968	113 ± 3	0.9975	1306 ± 167	0.9953
**16 nt**					0.9857	0.9974
**24 nt**	28 ± 0.8	0.9857	69 ± 2	0.9963	31 ± 0.5	0.9939

Figure S4. Titration measurements against RNAs by fluorescence anisotropy. All the experiments have been made in triplicate in 20 mM Tris-HCl pH 7.5, 5 mM β-mercaptoethanol (β-ME) and different salt concentrations (from 50 to 300 mM NaCl). The mixes were incubated 5 min at room temperature; (**a**) Binding titration of A/NP at 50 mM NaCl using 6-fluorescein amidite (FAM) alone; (**b**) Binding titration of A/NP and B/NP at 50, 150 and 300 mM NaCl using different length of short RNAs (from 5 to 24 nt); (**c**) Table of the different values obtained from the titrations.
